# Dynamic mass redistribution analysis of endogenous β-adrenergic receptor signaling in neonatal rat cardiac fibroblasts

**DOI:** 10.1002/prp2.24

**Published:** 2014-01-26

**Authors:** Rhonda L Carter, Laurel A Grisanti, Justine E Yu, Ashley A Repas, Meryl Woodall, Jessica Ibetti, Walter J Koch, Marlene A Jacobson, Douglas G Tilley

**Affiliations:** 1 Center for Translational Medicine, Temple University School of MedicinePhiladelphia, Pennsylvania, 19140; 2 Department of Pharmacology, Temple University School of MedicinePhiladelphia, Pennsylvania, 19140; 3 Moulder Center for Drug Discovery Research and Temple University School of PharmacyPhiladelphia, Pennsylvania, 19140

**Keywords:** Cardiac fibroblast, dynamic mass redistribution, epidermal growth factor receptor, fluorescence resonance energy transfer, β-adrenergic receptor

## Abstract

Label-free systems for the agnostic assessment of cellular responses to receptor stimulation have been shown to provide a sensitive method to dissect receptor signaling. β-adenergic receptors (βAR) are important regulators of normal and pathologic cardiac function and are expressed in cardiomyocytes as well as cardiac fibroblasts, where relatively fewer studies have explored their signaling responses. Using label-free whole cell dynamic mass redistribution (DMR) assays we investigated the response patterns to stimulation of endogenous βAR in primary neonatal rat cardiac fibroblasts (NRCF). The EPIC-BT by Corning was used to measure DMR responses in primary isolated NRCF treated with various βAR and EGFR ligands. Additional molecular assays for cAMP generation and receptor internalization responses were used to correlate the DMR findings with established βAR signaling pathways. Catecholamine stimulation of NRCF induced a concentration-dependent negative DMR deflection that was competitively blocked by βAR blockade and non-competitively blocked by irreversible uncoupling of Gs proteins. Subtype-selective βAR ligand profiling revealed a dominant role for β2AR in mediating the DMR responses, consistent with the relative expression levels of β2AR and β1AR in NRCF. βAR-mediated cAMP generation profiles revealed similar kinetics to DMR responses, each of which were enhanced via inhibition of cAMP degradation, as well as dynamin-mediated receptor internalization. Finally, G protein-independent βAR signaling through epidermal growth factor receptor (EGFR) was assessed, revealing a smaller but significant contribution of this pathway to the DMR response to βAR stimulation. Measurement of DMR responses in primary cardiac fibroblasts provides a sensitive readout for investigating endogenous βAR signaling via both G protein-dependent and –independent pathways.

## Introduction

Label-free technologies for investigation of ligand–receptor signaling responses have been increasingly used to explore the effects of distinct ligands on receptor-dependent signaling and to elucidate their mechanisms of action (Peters et al. 2010; Rocheville et al. 2013). The advantage of label-free technologies for the exploration of ligand-mediated effects on receptor signaling is that they provide an unbiased method to attain high throughput real-time kinetic signaling information and eliminate the need for use of multiple signal-specific assays that require intracellular dyes or overexpression of biomolecular reporters. Dynamic mass redistribution (DMR) is one such label-free technology that allows detection of the biological responses of cells to various stimuli via optical measurement of changes in reflected wavelength (Fang 2006). Deconvolution of the DMR responses with selective antagonists and uncouplers of G proteins reveals specific pathways that play a role in producing these effects (Schroder et al. 2011). Application of DMR technology has largely focused on characterizing G protein-coupled receptor (GPCR) signaling responses in a variety of clonal cell lines with either overexpressed or endogenous levels of receptors (Codd et al. 2011; Ferrie et al. 2011; Tran et al. 2012; Deng et al. 2013; Morse et al. 2013; Schrage et al. 2013). However, there exists a paucity of studies reporting endogenous GPCR DMR responses in primary cells (Schroder et al. 2010, 2011), which would enable investigators to better gauge the contribution of different receptor signaling pathways in response to ligand stimulation that may more closely reflect those in vivo.

β-adrenergic receptors (βAR) are important regulators of cardiac function under normal and pathologic conditions, and are expressed in both cardiomyocyte and cardiac fibroblast populations (Porter and Turner 2009). βAR can regulate numerous intracellular processes via both G protein-dependent and -independent mechanisms in a cell-type-specific manner. For instance, βAR can induce both cAMP synthesis (Gs protein-dependent) and epidermal growth factor receptor (EGFR) transactivation (G protein-independent), each of which can greatly impact cell function acutely and chronically (Tilley 2011). While much research has focused on defining the myriad βAR signaling pathways in cardiomyocytes, relatively fewer studies have explored the responses to βAR stimulation in cardiac fibroblasts, which have been shown to influence proliferation, survival, and the development of cardiac dysfunction during heart failure (Kim et al. 2002; Colombo et al. 2003; Turner et al. 2003; Cervantes et al. 2010; D'Souza et al. 2011; Jaffre et al. 2012). An agnostic approach to defining endogenous βAR signaling in cardiac fibroblasts would aid in interpreting which molecular pathways primarily drive the responses and may hold the most potential for intervention. Although the usefulness of DMR technology in detecting endogenous receptor signals in primary isolated cardiac cells has not been determined, it may provide a sensitive method to dissect βAR signaling pathways in cardiac fibroblasts with minimal manipulation. Thus, we utilized label-free whole cell dynamic mass DMR assays to measure endogenous βAR signaling in primary neonatal rat cardiac fibroblasts (NRCF) and dissect these responses with known antagonists of both G protein-dependent and-independent βAR signaling pathways.

## Material and Methods

### Materials

The following reagents were obtained from Sigma-Aldrich (St. Louis, MO): CGP 20712A (C231), cholera toxin (CTX) (C8052), dimethyl sulfoxide (DMSO) (D4540), dynasore (Dyn) (D7693), epidermal growth factor (E9644), ICI 118,551 (I127), Isoproterenol (I6504), propranolol (P0884), rolipram (Rol) (R6520), and salbutamol (S8260). Dobutamine (159780) was purchased from MP Biomedicals (Solon, OH) and gefitinib (Gef) (G-4408) from LC Laboratories (Woburn, MA).

### NRCF isolation and culture

NRCF were prepared from 1- to 2-day-old Sprague Dawley rat pups (Harlan Laboratories; Indianapolis, IN) by enzymatic digestion. Hearts were excised and placed in sterile albumin-dextrose-saline solution (116 mmol/L NaCl, 20 mmol/L 2-[4-(2-hydroxyethyl)piperazin-1-yl]ethanesulfonic acid (HEPES), 80 μmol/L Na2HPO4, 56 mmol/L glucose, 5.4 mmol/L KCl, 800 mmol/L MgSO4-7H2O; pH 7.35). After connective tissue and blood were removed, ventricles were minced and subjected to five 15-min enzymatic digestions using collagenase II (Worthington, Lakewood, NJ) and pancreatin (P3292, Sigma, St. Louis, MO). NRCF were harvested by plating on Nunclon™-treated 100 mm dishes (Nunc) for 2 h prior and subsequent removal of myocytes and dead cells. NRCF were cultured for 24 h in minimum essential media (10-010-CV; Corning/Cellgro, Corning, NY) containing 10% fetal bovine serum (FBS, 900-108; Gemini Bio-products, West Sacramento, CA) and 1% antibiotic–antimycotic solution (30-004-CI, Corning/Cellgro) at 37°C in a humidified incubator with 5% CO_2_. After 24 h, the media was replaced with 5% FBS-containing media. NRCF were maintained in culture for 3–14 days, as indicated.

### Dynamic mass redistribution

NRCF were trypsinized 2 days after primary isolation and using a BioTek Multiflo microplate dispenser were seeded at 1–2.5 × 10^4^ cells per well in Corning® Epic® 384 Well Fibronectin-Coated Cell Assay Microplates in 5% FBS-containing media. The DMR assay was performed 18 h later, corresponding to day 3 of cell culture, except in those experiments testing the impact of time in cell culture on DMR responsiveness, in which day 8 cells were used. When CTX was used, cells were treated at time of seeding in the 384-well plate for ∼16 h with 100 ng/mL CTX. Prior to DMR, the cells were rinsed and media was replaced with HBSS containing 20 mmol/L Hank's balanced salt solution and allowed to equilibrate for 1 h in the EPIC® Benchtop (BT) system (Corning®) at 37°C, as described by Schroder et al. (2011). Scan speed was 3 sec/scan with four scans/data point, therefore each data point was attained every 12 sec. Baseline DMR readings were attained for 5 min, after which antagonist (or buffer/DMSO control) additions occurred and readings continued for 30 min prior to agonist addition. Compound additions were performed using the Janus MDT liquid handler. DMR responses (change in pm shift) to agonist were normalized with corresponding buffer or antagonist additions. Each DMR (pm shift) point is presented as mean ± SEM and each experiment was performed in triplicate. Z′ was calculated using the methodology of Zhang et al. (1999) for isoproterenol (100 nmol/L) versus buffer control.

### Real-time qPCR

Total RNA was purified from NRCF cultured for 3–14 days using an RNeasy minikit following manufacturer's protocol (Qiagen, Valencia, CA). cDNA was synthesized using a High Capacity cDNA Reverse Transcription kit (Applied Biosystems [Life Technologies], Grand Island, NY), and real-time PCR (RT-PCR) was performed with SYBR® Select Master Mix (Applied Biosystems). Real-time PCR was performed using assay primers Rn00824536_s1 for *Adrb1* (β1AR), Rn00560650_s1 for *Adrb2* (β2AR), and Rn01775763_g1 for glyceraldehyde 3-phosphate dehydrogenase (GAPDH) at an annealing temperature of 60.0°C. Data from samples were analyzed in triplicate. All RT-PCR data were analyzed using Applied Biosystems Comparative CT Method (ΔΔCT) and βAR gene expression analysis was normalized to GAPDH. Data are presented as RQ values with RQ_min_ and RQ_max_ as error bars.

### Fluorescence resonance energy transfer detection of cAMP generation using ICUE3

ICUE3 adenovirus (Ad-ICUE3) was generously supplied by Dr. Yang Xiang (UC Davis). NRCF were seeded in the fibronectin-coated 10 mm glass-bottom insert of 35 mm dishes (MatTek Corporation, Ashland, MA) at 5 × 10^4^ cells/10 mm insert and infected with Ad-ICUE3 at a multiplicity of infection (MOI) of 40 for 24 h with or without CTX addition (100 ng/mL overnight). 24 h after infection, the cells were rinsed and media replaced with imaging buffer, as previously described (Tilley et al. 2010), prior to imaging using a Leica DMI4000B inverted microscope with a Leica DFC365 FX 1.4-megapixel monochrome digital camera with cyan fluorescent protein (CFP) excitation and CFP and yellow fluorescent protein (YFP) emissions measured every 2 sec. Cells were pretreated for 5 min with buffer or antagonists then, following 30 sec of baseline reads, the cells were stimulated with isoproterenol. The entire field-of view at 20× magnification was used to capture changes in fluorescence resonance energy transfer (FRET) in the NRCF population and each treatment condition was performed independently at least three times. Quantification of the ICUE3 ratio was calculated as changes in CFP emission/YFP emission over time, normalized to baseline.

### EGFR internalization

NRCF were seeded either in 35 mm glass-bottom dishes as described above or in clear-bottom black-walled 96-well plates (655090; Greiner Bio-One, Monroe, NC) at 3 × 10^4^ cells/well and infected with Ad-Flag-EGFR-mYFP (constructed at Vector Biolabs, Philadelphia, PA) at an MOI of 200 for 24 h prior to stimulation with agonist as indicated. After 1 h, cells were fixed in 4% paraformaldehyde (163201145; Wako Chemicals, Richmond, VA) for 20 min, rinsed with phosphate-buffered saline (Corning/Cellgro, 21-030-CV) and on-cell western staining (nonpermeabolized) was performed using anti-Flag M2 antibody (1:1000, 3 h at RT, Sigma, F1804), IRDye® 800CW Conjugated Goat (polyclonal) Anti-mouse secondary antibody (1:1000, 1 h at RT, LI-COR, 926-32210) and DRAQ5 (1:5000, 1 h at RT, Cell Signaling Technology, #4084) following the LI-COR on-cell protocol. Flag and DRAQ5 signals were detected using Odyssey CLx infrared imaging system (LI-COR Biosciences, Lincoln, NE) and YFP signal was measured with a Tecan M1000 plate reader. Amount of EGFR internalization was calculated by normalizing the Flag signal to both the DRAQ5 (cell number control) and YFP (infection control) signals: Flag/DRAQ5/YFP.

### Statistics

EC_50_ and *E*_max_ values were attained via nonlinear regression curve fitting of the DMR, ICUE3, or EGFR internalization responses using Prism 5.0 software (GraphPad Software Inc.; San Diego, CA). Statistical analyses were performed using a one-way analysis of variance (ANOVA) followed by a Newman–Keuls multiple comparisons test or a two-tailed unpaired *t*-test, as appropriate. A value of *P* < 0.05 was considered statistically significant.

## Results

### Optimization of cell culture conditions for detection of DMR responses to βAR stimulation in NRCF

**Figure 1:**
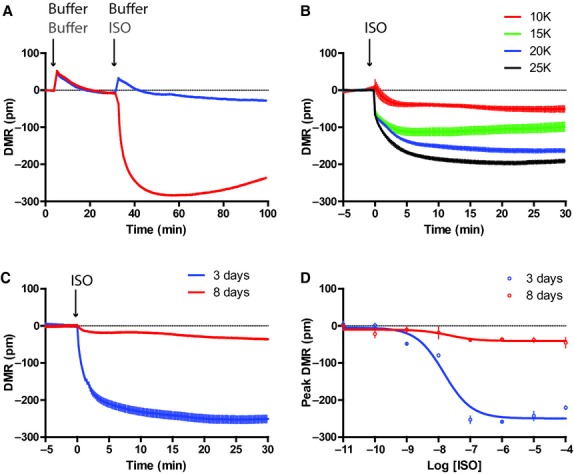
βAR stimulation-dependent DMR effects in NRCF. (A) Raw DMR traces showing two addition steps, the first (buffer) 5 min after establishing a baseline read of NRCF DMR and the second (ISO) 30 min after the first addition. Buffer additions cause transient positive DMR deflections (pm shift) that rapidly return to baseline and are subtracted as background from subsequent experiments. ISO (100 nmol/L) stimulation induced a rapid and sustained negative-deflected DMR response. Density was 2 × 10^4^cells/well. (B) Effect of cell plating density on βAR DMR response in NRCF. 1 × 10^4^−2.5 × 10^4^ cells/well were seeded and stimulated with 100 nmol/L ISO; 1 × 10^4^ and 1.5 × 10^4^ cells/well produced relatively small and variable DMR effects, while 2 × 10^4^ and 2.5 × 10^4^ cell/well produced more robust and consistent effects. Tracings are mean ± SEM (*n* = 3). (C) DMR responsiveness in 3 or 8 day NRCF cultures. ISO (100 nmol/L) produced a robust response in 3-day-old NRCF, an effect virtually absent in 8-day-old NRCF. Tracings are mean ± SEM (*n* = 3). (D) Concentration–response curves to ISO in 3- and 8-day NRCF reveal significantly reduced βAR responsiveness in 8-day-old cells (*E*_max_ = −40 ± 5.3 pm) versus 3-day-old cells (*E*_max_ = −250 ± 8.2 pm). Data are mean ± SEM, *n* = 3 per concentration point.

To determine if βAR stimulation of NRCF produces a detectable DMR response, we stimulated the cells with either buffer or isoproterenol (ISO, 100 nmol/L). Examples of single raw DMR response traces in response to buffer or ISO are shown in Figure 1A, where background subtractions have not been performed. In subsequent experiments, the buffer alone treatments (± appropriate antagonist treatments) served as background effects of drug additions and were subtracted from the drug treatment responses. ISO produced a large negative DMR response, well separated from the buffer control and producing a Z′ of 0.8, indicating an excellent signal range for detection with low variability in this assay (Zhang et al. 1999). As cell density has been reported to impact the magnitude of DMR response (Fang 2006), the impact of seeding increasing numbers of NRCF in the 384-well plates (1 × 10^4^ − 2.5 × 10^4^ per well) on the ISO-mediated DMR response was evaluated. NRCF seeded at 1.5 × 10^4^ and 1 × 10^4^ per well displayed markedly reduced responses of ∼50% and 25% of the response attained with 2.5 × 10^4^ cells, respectively, and with high variability (Fig. 1B). However, NRCF seeded at 2.5 × 10^4^ and 2 × 10^4^ per well displayed comparable negative DMR responses to ISO with very low variation among replicates, thus 2 × 10^4^ cells per well were utilized for subsequent testing.

Primary NRCF can be maintained in culture for days to weeks, however, their phenotype becomes altered toward a myofibroblast phenotype over time (Santiago et al. 2010), and therefore, we tested whether the DMR response to ISO stimulation is preserved during cell culture. NRCF cultured for 3 or 8 days were stimulated with ISO. Although the 3-day-old NRCF showed robust negative DMR response to ISO, this response was almost completely absent in the 8-day-old NRCF (Fig. 1C). In fact, βAR responsiveness was almost abolished in the 8-day-old cells versus the 3-day-old cells over a range of ISO concentrations (Fig. 1D), therefore all subsequent experiments were performed in 3-day-old NRCF.

### βAR subtype-selective impact on DMR responses to catecholamine stimulation on NRCF

**Figure 2:**
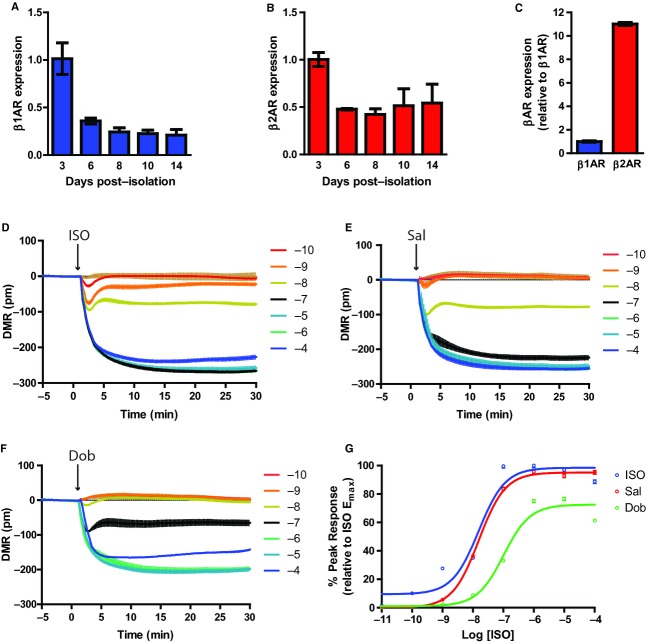
βAR subtype-selective expression and DMR responsiveness in primary NRCF. Real-time PCR data show a rapid loss (≥50%) of β1AR (A) and β2AR (B) expression following 3 days of cell culture and demonstrate ∼11-fold higher expression of β2AR than β1AR in 3-day-old primary NRCF (C). Data expressed as RQ values with RQ_min_ and RQ_max_ values as error. DMR responses (pm shift) in response to ISO (β1AR/β2AR nonselective, D), salbutamol (Sal, β2AR-selective, E), and dobutamine (Dob, β1AR-selective, F) in a concentration-dependent manner. Tracings are mean ± SEM (*n* = 3). (G) Summarized concentration–DMR response curves for ISO, Sal, and Dob. Data are expressed as % of peak ISO response; mean ± SEM, *n* = 3 per concentration point.

The loss in DMR responsiveness to ISO with prolonged culture of NRCF correlates with a decrease in both β1AR and β2AR gene expression (Fig. 2A and B), where a dramatic loss in expression of each is observed after day 3 in culture. Although β1AR expression in cultured NRCF was detected, the loss of DMR responsiveness to ISO is most likely due to the decreased β2AR expression, as β2AR gene expression at day 3 was observed to be 11-fold greater than that of β1AR (Fig. 2C), congruent with previous studies reporting the dominant expression and activity of β2AR in NRCF (Lau et al. 1980; Gustafsson and Brunton 2000; Yin et al. 2003; Cervantes et al. 2010). DMR responses to treatment of NRCF with increasing concentrations of βAR subtype-selective agonists (ISO [β1/β2 nonselective, Fig 2D], salbutamol [Sal, β2-selective, Fig. 2E], and dobutamine (Dob, β1-selective, Fig. 2F]) were measured. Increasing concentrations of each agonist lead to increasingly negative DMR deflections. For ISO concentrations up to 1 nmol/L, the DMR response was transient, returning close to baseline within 5 min of stimulation, whereas at 10 nmol/L, the DMR response showed two phases, an initial downward deflection that recovered toward baseline slightly and a more prolonged negative DMR. At concentrations of 100 nmol/L and higher, the negative DMR response was rapid and sustained, achieving maximal deflection. Sal produced similar DMR responses to ISO, whereas Dob was only able to induce a maximal DMR response of ∼75% of the ISO and Sal responses at micromolar concentrations. In all, the βAR subtype-selective agonist responses are also in agreement with the expression data above, where Sal produced a nearly identical concentration–DMR response curve to that of ISO, while Dob had a markedly reduced maximal DMR effect and rightward-shifted potency for producing the DMR effect (Fig. 2G). EC_50_ and *E*_max_ data for each ligand are shown in Table 1.

**Table 1:** EC_50_ and *E*_max_ DMR data for each βAR ligand

Ligand	EC_50_ (nmol/L)	*P* value vs. buffer	*E*_max_ (pm)	*P* value vs. buffer
Isoproterenol +				
Buffer	15.4 ± 4.2	–	−266.1 ± 8.2	–
Cholera toxin (100 ng/mL)	30.1 ± 17.4	ns	−109.6 ± 6.4	<0.001
Propranolol (1 μmol/L)	808.8 ± 84.1	<0.001	−247.6 ± 4.3	<0.05
Salbutamol +				
Buffer	15.6 ± 0.8	–	−257.1 ± 1.8	–
Cholera toxin (100 ng/mL)	30.1 ± 4.9	ns	−122.4 ± 2.1	<0.001
Propranolol (1 μmol/L)	1,9710 ± 1749	<0.001	−251.9 ± 6.3	ns
Dobutamine +				
Buffer	133.8 ± 11.7	–	−217.1 ± 3.5	–
Cholera toxin (100 ng/mL)	50.0 ± 16.8	<0.001	−86.0 ± 3.4	<0.001
Propranolol (1 μmol/L)	nc	–	nc	–
Propranolol	32.7 ± 6.4	–	81.2 ± 2.5	–
ICI 188,551	192.1 ± 61.7	–	117.3 ± 5.4	–
CGP 20712A	nc	–	nc	–

*P* values attained via one-way ANOVA with Newman–Kewls multiple comparison post hoc test within each treatment group. ns, not significant; nc, data not converged.

**Figure 3:**
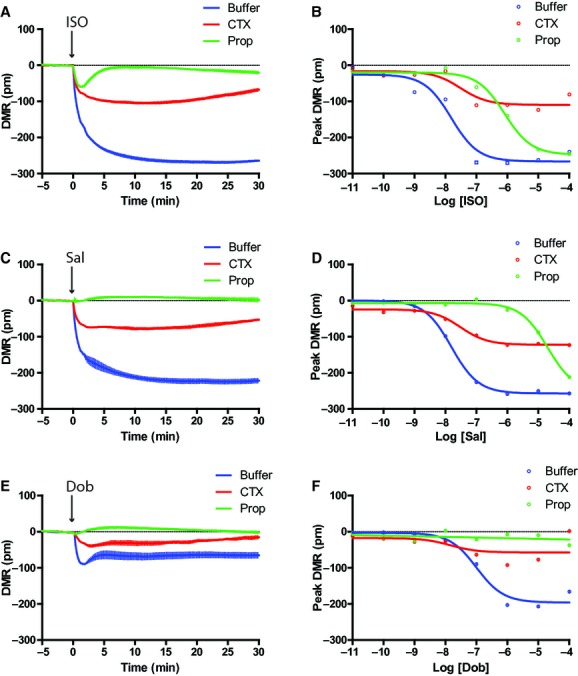
βAR-mediated DMR responses are primarily Gs protein-dependent. (A) Pretreatment of NRCF with either CTX (100 ng/mL overnight) or propranolol (Prop, 10 μmol/L for 30 min) decreased ligand-dependent DMR responses (pm shift) induced by ISO (A), Sal (C), and Dob (E), at 100 nmol/L each. Tracings are mean ± SEM (*n* = 3). Analysis of the concentration-dependent effect of each ligand on NRCF DMR response in the presence or absence of the inhibitors showed a classic competitive inhibition by Prop and noncompetitive inhibition by CTX for ISO (B), Sal (D), and Dob (F). Inhibition of G protein-dependent DMR effects by CTX accounts for a majority of the responses to βAR ligands. Data are mean ± SEM, *n* = 3 per concentration point.

Next, deconvolution of the DMR signal in response to βAR ligands was measured via standard βAR blockade with propranolol (Prop, 1 μmol/L) and depletion of canonical Gs protein activity with CTX (100 ng/mL). CTX irreversibly ADP-ribosylates Gs protein to enhance its degradation and diminish its biochemical activity over several hours (Chang and Bourne 1989), and has been used in other studies to assess the contribution of Gs protein activity to DMR responses (Schroder et al. 2010, 2011; Ferrie et al. 2013). Consistent with its reversible antagonism of βARs, Prop competitively inhibited the DMR responses to ISO (Fig. 3A and B) and Sal (Fig. 3C and D), producing rightward shifts in concentration–response curves and reducing the potency of each ligand (Table 1). CTX noncompetitively blocked ISO and Sal responses, thereby not altering their potencies, but dramatically reducing their maximal DMR effects (Fig. 3A–D, Table 1). As observed above, Dob produced blunted DMR responses compared to either ISO or Sal, which were completely blocked by Prop and substantially reduced by CTX (Fig. 3E and F, Table 1). The Dob-induced DMR responses at high concentrations (at or above 1 μmol/L) are consistent with its reported affinity for activation of β2AR and known lower efficacy compared with ISO or Sal (Baker 2010).

**Figure 4:**
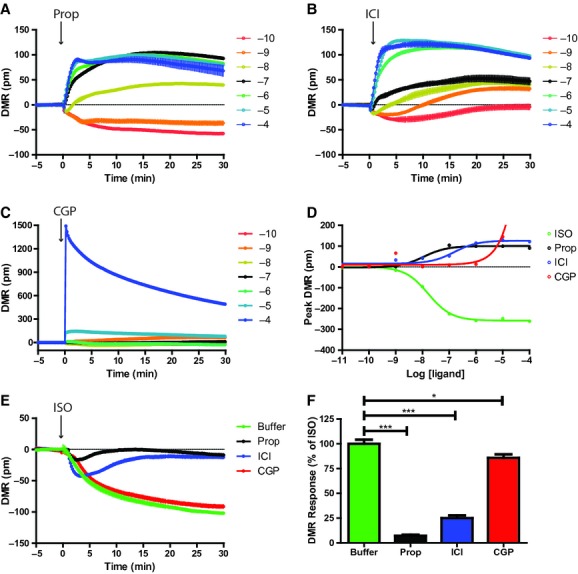
βAR-mediated DMR responses in NRCF are primarily driven through β2AR stimulation. DMR responses to increasing concentrations of βAR antagonists were measured revealing that lower concentrations of either propranolol (Prop, β1AR/β2AR nonselective, A) or ICI 118,551 (ICI, β2AR-selective, B) induced slightly negative deflections, while at higher concentrations induced positive deflections, consistent with their reported inverse agonist activities at β2AR. CGP 20712A (CGP, β1AR-selective, C) did not induce changes in DMR until 10 μmol/L. Tracings are mean ± SEM (*n* = 3). (D) Summary of the concentration-dependent effects on DMR responses in NRCF of Prop, ICI, and CGP compared with ISO. Data are mean ± SEM, *n* = 3 per concentration point. (E) ISO (100 nmol/L)-induced DMR responses were tested alone or in conjunction with 10 μmol/L of Prop, ICI, or CGP, revealing that Prop and ICI produced the greatest inhibition of ISO-induced DMR, while CGP had only a small impact on the response. Tracings are mean ± SEM (*n* = 3). (F) Summary of data in (E) via nonlinear regression analysis of the peak DMR responses, expressed as % of the ISO response. Data are mean ± SEM (*n* = 3); *P < 0.05, ***P < 0.001, ANOVA.

To further support β2AR subtype-dependent signaling in NRCF, we also assessed the DMR response of βAR antagonists with differing selectivity: Prop (β1/β2 nonselective), ICI 118,551 (ICI, β2-selective) and CGP 20712A (CGP, β1-selective), either alone or in conjunction with ISO. Prop and ICI have been previously reported to act as inverse agonists in several cell types (Azzi et al. 2003; Taira et al. 2010), and indeed here, we show that each concentration dependently induces progressively positive-deflected DMR responses (Fig. 4A and B). Although the cause of the negative-deflected DMR responses at 100 pmol/L and 1 nmol/L Prop are unknown, the progressively positive-deflected DMR responses attained with increasing Prop concentrations beginning at 1 nmol/L are consistent with previous studies wherein Prop began to induce inverse β2AR activity at a concentration as low as 1 nmol/L (Chidiac et al. 1994; Azzi et al. 2001). Inverse agonism produced by CGP has also been reported (Engelhardt et al. 2001; Janssens et al. 2008), though in our assay only induced a rapid positive deflection in DMR at 10 μmol/L (Fig. 4C); CGP at 100 μmol/L produced an extreme positive DMR deflection that is likely due to a toxic effect on the cells and was excluded from further analysis. A comparison of the concentration-dependent effects of the βAR antagonists on the DMR response with that of ISO is shown in Figure 4D and potency and efficacy data in Table 1. Each of the βAR antagonists induced a positive-deflected DMR response in NRCF to a similar extent at 10 μmol/L, and this concentration of each was next used to determine their ability to block ISO (100 nmol/L)-induced responses, with appropriate antagonist alone background subtractions performed as described in methods. As shown in Figure 4E and F, both Prop and ICI very significantly reduced the ISO-mediated DMR effect, while CGP produced a very small, though significant, decrease in the ISO response, again confirming a dominant role for β2AR signaling in NRCF.

### DMR responses to catecholamine in NRCF primarily reflect activation of Gs protein/cAMP-dependent signaling

**Figure 5:**
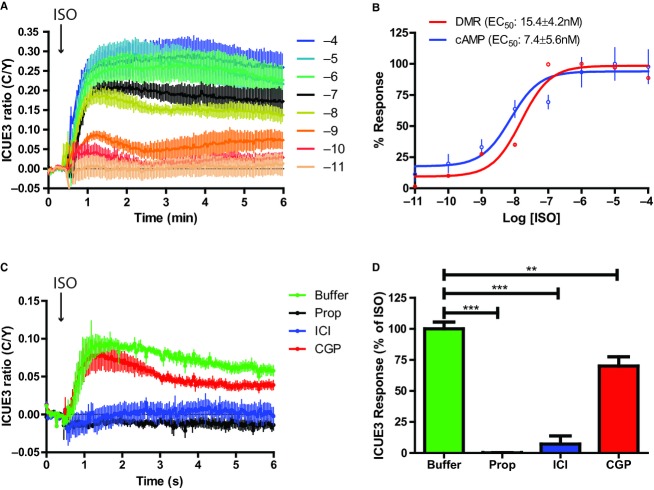
ISO stimulates similar cAMP generation and DMR responses in NRCF. (A) Averaged ICUE3 ratio responses to increasing concentrations of ISO; tracings are mean ± SEM, *n* = 3–6 independent dishes of NRCF infected with ICUE3 for each concentration of ISO. (B) Concentration–response curves comparing ICUE3 and DMR data. Each assay produced similar potencies for ISO-mediated effects. Data are mean ± SEM, *n* = 3–6 per concentration point. (C) Timecourse of cAMP generation response to ISO (1 μmol/L) in the presence or absence of 10 μmol/L Prop, ICI, or CGP. Tracings are mean ± SEM (*n* = 3–4). (D) Comparison of inhibitor sensitivity of ISO ICUE3 responses in NRCF, expressed as % of ISO response. Data are mean ± SEM (*n* = 3–4); **P < 0.01, ***P < 0.001, ANOVA.

To determine if the DMR responses observed in NRCF could be validated via an independent approach, we utilized a fluorescence biosensor for cAMP (Indicator for cAMP Using EPAC1, ICUE3 [Allen et al. 2006]) to measure cAMP generation in the NRCF via detection of changes in FRET (Fig. S1A). ISO stimulation concentration dependently increased cAMP generation in the cells, revealing similar kinetics to those of the DMR responses, with lower concentrations producing transient responses and higher concentrations producing prolonged responses (Fig. 5A). Of note, although the variability within replicates was higher in the cAMP FRET assay versus the DMR assay, the concentration–response curve fits for the DMR and cAMP assays were almost superimposable and produced similar EC_50_ values (Fig. 5B). We next determined how βAR subtype-selective antagonists impacted ISO-mediated cAMP generation compared with those observed via DMR. Indeed, ISO-induced FRET was significantly blocked by both Prop and ICI, whereas CGP produced a partial blockade (Fig. 5C and D), further supporting that β2AR-dependent signaling predominates in NRCF. Interestingly, and in contrast to the DMR responses, despite Prop alone producing a small reduction in FRET, the cAMP FRET signals were not impacted by the antagonists alone (Fig. S1B–D), again demonstrating the sensitivity of DMR for detection of less robust effects of ligands on biological responses.

**Figure 6:**
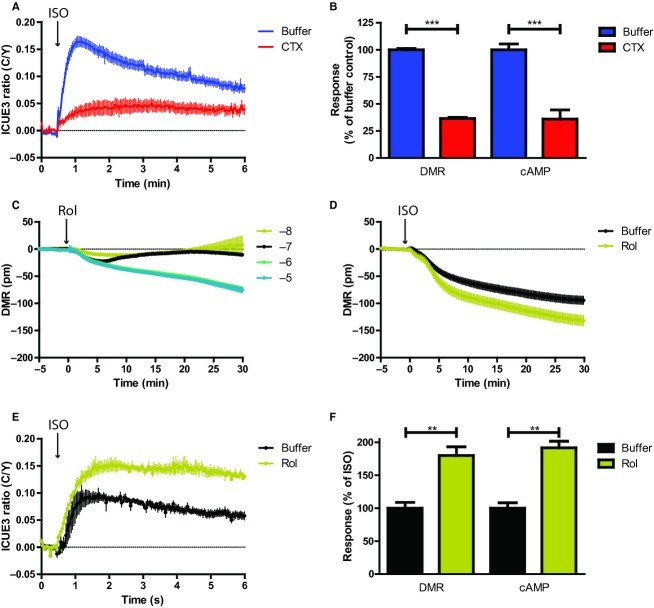
Changes in cAMP levels predict βAR-dependent DMR responses. (A) ISO (1 μmol/L)-mediated cAMP generation is greatly reduced in the presence of CTX (100 ng/mL overnight), to a similar extent as the CTX-dependent decrease in DMR response. Tracings are mean ± SEM (*n* = 4). (B) Summary of the impact of CTX on ISO-mediated cAMP and DMR effects in NRCF; data are mean ± SEM (*n* = 3–4), ***P < 0.001, two-tailed *t*-test. (C) Rolipram (Rol, PDE4 inhibitor) produced negative DMR deflections at 1 and 10 μmol/L, with much less impact at 10 and 100 nmol/L. Rol (10 nmol/L) significantly enhanced the ISO-mediated DMR (D) and ICUE3 (E) responses. Tracings are mean ± SEM (*n* = 3). (F) Summary of peak DMR and cAMP responses from (D) and (E), expressed as % of ISO effect; data are mean ± SEM (*n* = 3), **P < 0.01, two-tailed *t*-test.

As a majority of the DMR response to βAR stimulation in NRCF was shown in Figure 3 to be Gs protein-dependent, we sought to determine if this could be recapitulated via the cAMP FRET assay. ISO-dependent cAMP generation was substantially reduced by pretreatment of the cells with CTX (Fig. 6A). Quantitation of the impact of CTX on both DMR and cAMP responses to ISO revealed an identical reduction of effects in both assay readouts (Fig. 6B). If cAMP-dependent signaling comprises the majority of the endogenous βAR DMR response in NRCF, we posited that inhibition of the degradation of cAMP by phosphodiesterases (PDE) should enhance the negative DMR response to ISO. To test this, we used Rol, an inhibitor of the PDE4 family, known to be directly involved in the desensitization to βAR signaling (Fu et al. 2013). Alone, Rol concentration dependently induced a negative-deflected DMR response (Fig. 6C), suggesting that a pool of PDE4-sensitive cAMP is generated basally under DMR assay conditions in these cells, with 10 nmol/L Rol producing the smallest alteration in DMR. Using this concentration of Rol in conjunction with ISO, it was observed that PDE4 inhibition does indeed enhance the ISO-induced DMR response (Fig. 6D), which was also confirmed via the cAMP FRET assay (Fig. 6E and F). Others have reported that inhibition of receptor internalization may also impact GPCR DMR signatures (Fang et al. 2005). Interestingly, using an inhibitor of dynamin (Dyn), we observed an enhanced ISO-mediated negative DMR response, despite inducing a positive-deflected DMR response on its own at concentrations close to above its known IC_50_ of 15 μmol/L (Kirchhausen et al. 2008) (Fig. S2A–C). Thus, consistent with βAR downregulation playing a role in desensitization of acute Gs protein-dependent signaling, inhibition of receptor internalization increases the G protein-dependent DMR response to ISO in NRCF.

### EGFR-dependent signaling contributes to the DMR response to βAR stimulation in NRCF

**Figure 7:**
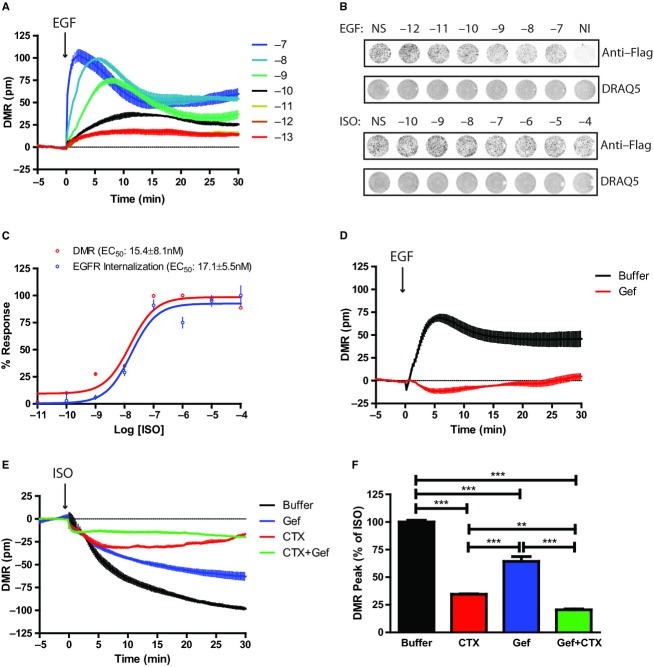
The βAR-dependent DMR response is sensitive to inhibition of EGFR signaling in NRCF. (A) EGF concentration dependently induces a positive-deflected DMR response in NRCF. Tracings are mean ± SEM, *n* = 3. (B) EGF and ISO each concentration dependently induce Flag-EGFR-mYFP internalization as detected via a modified on-cell western assay in NRCF. Anti-Flag immunofluorescence was normalized to both DRAQ5 (nuclear stain) and mYFP fluorescence (infection control), as described in methods. Data shown are representative of on-cell assay results. (C) Comparison of concentration-dependent ISO-mediated EGFR internalization and DMR responses reveal similar potencies for effects. Data are mean ± SEM, *n* = 3 per concentration point. (D) Pretreatment of NRCF with the EGFR antagonist gefitinib (Gef, 1 μmol/L, 30 min) blocked the EGF (1 nmol/L)-induced DMR response. Tracings are mean ± SEM (*n* = 3). (E) Pretreatment of NRCF with either CTX (100 ng/mL overnight) or Gef (1 μmol/L) reduced the ISO (100 nmol/L)-mediated DMR response, with a further reduction produced by the combination of both CTX and Gef. Tracings are mean ± SEM (*n* = 3). (F) Summary of DMR peak responses from (E), expressed as % of ISO effect, where CTX and Gef given alone reduced the ISO response by ∼65% and ∼35%, respectively, whereas a combination of CTX + Gef reduced the ISO DMR response by ∼80%. Data are mean ± SEM (*n* = 3); **P < 0.01, ***P < 0.001, ANOVA.

While G protein-dependent signaling clearly plays a dominant role in the DMR response to βAR stimulation in NRCF, we also aimed to assess whether G protein-independent βAR signaling could be detected using this technology. The most well-recognized G protein-independent cellular effects are engaged via G protein-coupled receptor kinase (GRK)/β-arrestin-mediated signaling, which impact numerous processes including receptor internalization, formation of intracellular signaling scaffolds, and transactivation of EGFR, as well as cardiac outcomes such as hypertrophy and survival (Tilley 2011). As we showed that inhibition of receptor internalization enhanced G protein-dependent βAR signaling (Fig. S2A–C) and direct interference of GRK/β-arrestin signaling would promote a similar effect, we assessed the contribution of EGFR signaling, a well-characterized downstream readout of G protein-independent βAR signaling (Maudsley et al. 2000; Noma et al. 2007; Tilley et al. 2009), to the observed βAR-mediated DMR response. To first establish whether NRCF produce EGFR-dependent DMR responses, we measured the effect of EGF on the cells, which induced concentration-dependent positive deflected DMR responses (Fig. 7A), with an EC_50_ of less than 1 nmol/L (Fig. S2D). To ensure that βAR stimulation induces EGFR activation in NRCF, we infected the cells with adenovirus containing Flag- and mYFP-tagged EGFR, which becomes internalized upon ligand stimulation (Fig. S2E). Using a nonpermeabolized on-cell western assay to measure loss of cell-surface Flag-epitope upon ligand stimulation, we assessed receptor internalization responses to ISO, with EGF as a positive control (Fig. 7B). Each ligand induced EGFR internalization, with EGF producing more efficacious and potent receptor internalization than ISO (Fig. S2F), as previously reported (Tilley et al. 2009). Comparison of the concentration–response curves between ISO-induced DMR and EGFR internalization responses revealed almost identical EC_50_ values for each effect (Fig. 7C). To determine if EGFR signaling contributes to the βAR DMR response, we next pretreated the NRCF with the EGFR antagonist Gef, which completely blocks the EGF-dependent DMR response (Fig. 7D). In cells pretreated with Gef, the ISO-induced DMR response was significantly reduced to ∼65% of the buffer-pretreated ISO response (Fig. 7E and F). We compared this response to that produced by ISO in the presence of CTX, which reduced the DMR response to ∼35% of the ISO-mediated response. Separately, these results indicate that the CTX- and Gef-sensitive ISO responses comprise all of the βAR-dependent DMR response, however, combined treatment of NRCF with both CTX and Gef did not result in complete ablation of the ISO-mediated response, instead reducing it to ∼20% (Fig. 7E and F). These results may suggest that CTX- and Gef-sensitive βAR DMR responses overlap, perhaps converging upon a shared pathway(s), and that ∼20% of the βAR DMR response is both G protein- and EGFR-independent.

## Discussion

Although label-free detection of receptor responses to stimulation via DMR analysis has been utilized for several years, few studies have investigated the usefulness of this assay for dissecting endogenous receptor biology in primary cells. Two recent studies by the Kostenis group showcased the DMR response to GPCR ligands in primary human keratinocytes and neutrophils (Schroder et al. 2010, 2011), but otherwise cultured cell lines have been favored for investigation of receptor signaling effects. Here, we used DMR to assess the utility of label-free assays to detect and explore biological responses to receptor stimulation in primary cardiac fibroblasts, with a focus on βAR ligand-mediated signaling effects through endogenously expressed β2AR. The sensitivity for ligand-mediated DMR effects was excellent and the intraexperimental variability low, although we did observe that maximal DMR effects attained between distinct primary NRCF preparations varied in response to ISO. The variation in response to βAR stimulation in the primary cell preparations was also observed in the ICUE3 cAMP detection assay, and therefore, we conclude that it is a result of variability in the primary cell preparation methods and not the assay detection methods. Altogether, our results demonstrate that measurement of DMR in response to βAR stimulation with a variety of ligands in NRCF provides very sensitive detection of agonist and inverse agonist activities, distinguishes the contribution of canonical Gs protein-dependent versus -independent βAR signaling, and highlights important assay conditions for the use of primary NRCF for DMR detection including cell density and postisolation time in culture.

Other cell types have displayed either positive or negative DMR responses to stimulation of GPCR (Schroder et al. 2010), and β2AR in particular (Fang and Ferrie 2008; Ferrie et al. 2011, 2013; Lamyel et al. 2011; Stallaert et al. 2012). Here, we observed a substantial negative DMR response following βAR stimulation in NRCF. The overall DMR kinetics of βAR stimulation in NRCF differed from those detected in A431 clonal cells, which have been used extensively to study β2AR-mediated DMR (Fang and Ferrie 2008; Ferrie et al. 2013), where a rapid and transient negative DMR response typically precedes a slower, larger, and sustained positive DMR response. In primary NRCF, we observed a biphasic negative DMR response consisting of an acute transient deflection followed by a prolonged phase that slowly decayed toward baseline at ligand concentrations <100 nmol/L, whereas a rapid and robust negative DMR was sustained at concentrations >100 nmol/L, which had greater similarity to DMR responses to β2AR stimulation observed in MRC-5 human-derived lung fibroblasts (Lamyel et al. 2011). The observed separation of βAR-mediated DMR effects in NRCF into low and high ligand concentration-sensitive G protein-dependent signaling patterns is consistent with previous studies of βAR DMR and cAMP responses to stimulation. With regard to DMR, Ferrie et al. (2013) demonstrated that β2AR stimulation in A431 cells produced a rapid early wave of signaling that was achieved with lower ligand concentrations acting at membrane-localized β2AR. Higher ligand concentrations produced a slower sustained wave of intracellular-localized β2AR signaling that was still G protein-dependent, consistent with recent study highlighting the ability of internalized β2AR to continue signaling via activated Gs protein in HEK 293 cells (Irannejad et al. 2013).

This ligand concentration-dependent effect on βAR-G protein signaling has also been demonstrated in cardiomyocytes where lower βAR ligand concentrations produce local cAMP signaling effects that are buffered tightly by PDE4 (De Arcangelis et al. 2010), whereas higher ligand concentrations can overcome PDE4 regulation to induce greater cAMP production and more widespread intracellular signaling, as recently reviewed (Fu et al. 2013). Similarly, the rapid and transient nature of the DMR response to lower concentrations of βAR ligand in NRCF was also observed in our cAMP assays and was enhanced by inhibition of PDE4. Interestingly, inhibition of dynamin-dependent receptor internalization enhanced the βAR ligand-mediated DMR response in NRCF, which has also been shown for β2AR in A431 cells (Goral et al. 2011). This suggests that although prolonged intracellular β2AR-Gs protein-mediated signaling occurs, the acute membrane-localized DMR response is negatively regulated through receptor internalization processes.

The majority of the DMR signal detected in response to βAR stimulation in NRCF involved Gs protein-dependent cAMP generation, highlighted by both the enhanced negative DMR response attained in conjunction with PDE4 inhibition and the significant reduction in response by Gs protein inhibition with CTX. The CTX-sensitive DMR response to βAR stimulation was observed at early and late time points and at low and high concentrations of βAR ligand, consistent with both rapid Gs protein signaling at the membrane and persistent intracellular Gs protein activity at high ligand concentrations. Of note, however, CTX did not ablate all cAMP generation as detected in the cAMP FRET assay, indicating incomplete inhibition of Gs protein, which has also been observed by others (Stallaert et al. 2012). The residual ISO-induced cAMP generation in the presence of CTX suggests that the DMR effects observed may represent significantly reduced, but not completely ablated Gs protein signaling. Thus, the proportion of Gs protein-dependent signaling that comprises the measured βAR DMR effects may actually be slightly greater than estimated by the data.

Despite the majority of the βAR ligand-induced DMR signal being mediated via Gs protein-dependent effects, G protein-independent effects could also be extrapolated from this assay through the following key observations. First, CTX pretreatment of the NRCF completely blocked the DMR response to low concentrations (<100 nmol/L) of either ISO or Sal, consistent with lower βAR ligand concentrations activating primarily rapid and transient membrane-associated Gs protein-dependent signaling. Second, although βAR ligand concentrations >100 nmol/L had reduced DMR responses in the presence of CTX, they still induced immediate responses that were persistent throughout the study, which is also consistent with the previously reported rapid non-G protein-dependent signaling wave observed at higher βAR ligand concentrations in A431 cells (Ferrie et al. 2013). Lastly, inhibition of a well-characterized distal G protein-independent βAR signaling process, EGFR transactivation (Maudsley et al. 2000; Kim et al. 2002; Noma et al. 2007; Tilley et al. 2009), resulted in a significant reduction in ISO-mediated DMR in the NRCF. As EGF induced a positive DMR response, we surmised that βAR-dependent EGFR transactivation could also promote a positive DMR response that would be masked by the overall negative DMR effect induced by ISO. We therefore predicted that inhibiting EGFR would block any hidden positive DMR response and enhance the negative DMR response to ISO. However, Gef pretreatment of the NRCF, either alone or in conjunction with CTX, resulted in a reduced negative DMR effect following ISO stimulation. This result suggests that βAR-mediated EGFR transactivation may induce a different DMR signature than direct EGFR stimulation, an idea consistent with our previous study showing that differential activation of EGFR, via βAR-dependent transactivation versus direct ligand stimulation, results in distinct downstream signaling events (Tilley et al. 2009).

Thus, the balance between Gs protein-dependent and-independent signaling effects in response to stimulation of endogenous β2AR in primary cardiac fibroblasts is primarily routed through Gs protein (≥65%), while the remainder (≤35%) is Gs protein-independent, including a component of the DMR response mediated via EGFR transactivation (≤15%). The residual Gs protein/EGFR-independent portion of the DMR response may be mediated via other mechanisms including GRK/β-arrestin-dependent signaling. This portion of the DMR effect may be challenging to study as GRK/β-arrestin regulation of GPCR signaling is a well-documented mechanism of receptor desensitization (Moore et al. 2007), thus inhibition or deletion of these components of βAR signaling could augment the Gs protein-dependent DMR response. Biased ligands selective for Gs protein-independent pathways may provide a useful tool for studying such paradigms, and indeed β-blockers that have been shown to be biased for β-arrestin-dependent signaling (carvedilol and alprenolol) have been demonstrated to produce long-lasting DMR effects with a similar deflection as unbiased βAR agonists (Ferrie et al. 2011). That the Gs protein-independent DMR signature in this study, as well as in ours, persists from acute to longer time points suggests an important impact of G protein-independent pathways chronically, which indeed has been demonstrated for other GPCR from the cellular level to large animal studies (Violin et al. 2010; Boerrigter et al. 2011; Kim et al. 2012). Therefore, DMR analysis of endogenous receptor systems will provide a useful approach to define the contribution of such signaling mechanisms to the biological responses to differential ligand stimulation and relate these responses to functional outcomes in primary cells.

## Abbreviations

ANOVA: analysis of variance
CTX: cholera toxin
DMR: dynamic mass redistribution
Dob: dobutamine
Dyn: dynasore
EGFR: epidermal growth factor receptor
FBS: fetal bovine serum
FRET: fluorescence resonance energy transfer
GAPDH: glyceraldehyde 3-phosphate dehydrogenase
Gef: gefitinib
GPCR: G protein-coupled receptor
GRK: G protein-coupled receptor kinase
ISO: isoproterenol
MOI: multiplicity of infection
NRCF: neonatal rat cardiac fibroblasts
PDE: phosphodiesterases
Rol: rolipram
βAR: β-adenergic receptors

## Supporting Information

**Figure S1.** βAR antagonist impact on cAMP generation in NRCF. (A) Ad-ICUE3 expression and detection in primary NRCF before (left) and 90 sec after (right) ISO (1 μmol/L) addition at 30 sec (20× magnification, CFP excitation, YFP emission); increased cAMP generation in response to ISO decreases YFP emission, right panel. βAR-selective antagonist impact on cAMP generation was assessed via ICUE3 assay, where only very small effects of Prop (B), ICI (C), and CGP (D) were detected. Tracings are mean ± SEM (*N* = 3).

**Figure S2.** Dynasore and EGF signaling responses in NRCF. (A) The dynamin inhibitor dynasore (Dyn) had little impact on DMR responsiveness in NRCF at 1 μmol/L, but at or above its reported IC_50_ of 15 μmol/L, it induced positive DMR deflections. (B) Dyn pretreatment (100 μmol/L, 30 min) enhanced the ISO-mediated DMR effect. Tracings are mean ± SEM (*n* = 3). (C) Summary of peak ISO-induced DMR response from (B). Data are mean ± SEM (*n* = 3). **P* < 0.05, two-tailed *t*-test. (D) EGF concentration dependently induced a positive-deflected DMR response in NRCF with an EC_50_ < 1 nmol/L. Data are mean ± SEM, *n* = 3 per concentration point. (E) EGF (1 nmol/L; 30 min treatment) induces the internalization of adenovirally expressed Flag-EGFR-mYFP in NRCF. Green = EGFR, blue = DRAQ5 nuclear stain. (F) Summary of on-cell assay for loss of surface Flag-EGFR-mYFP in response to increasing concentrations of EGF and ISO treatment for 60 min. Data are mean ± SEM, *n* = 3 per concentration point.
