# Supplementary figures and images for: Dynamic mass redistribution analysis of endogenous β-adrenergic receptor signaling in neonatal rat cardiac fibroblasts
Source: Pharmacol Res Perspect. 2014 Jan 26;2(1):e00024. doi: 10.1002/prp2.24 (PMC3968527; doi:10.1002/prp2.24)

A

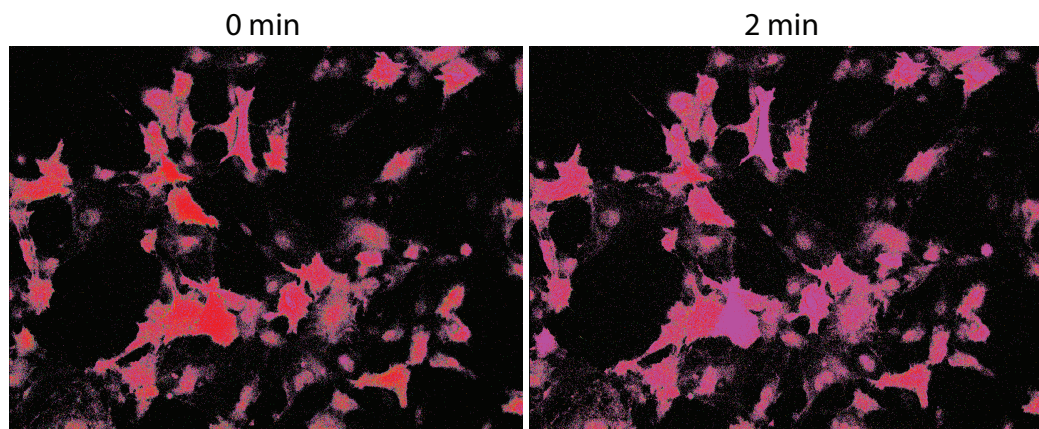

B

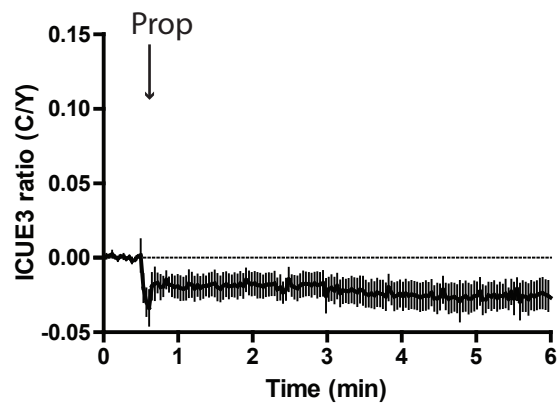

C

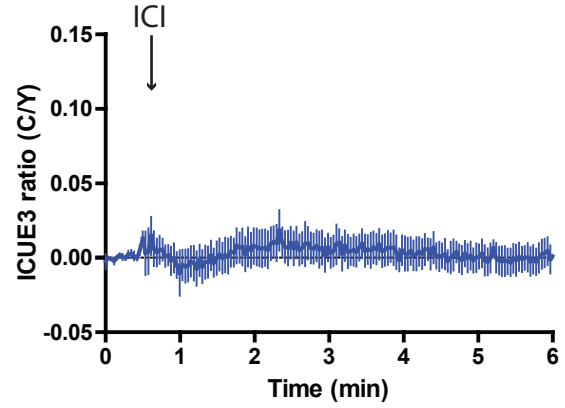

D

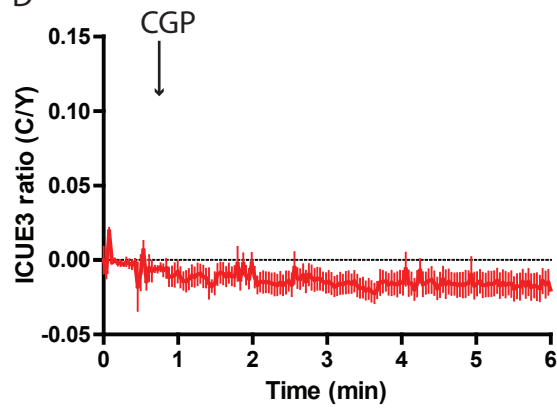

Supplemental Figure 1

Supplement: Supplementary file 1 — Figure S1. βAR antagonist impact on cAMP generation in NRCF. (A) Ad-ICUE3 expression and detection in primary NRCF before (left) and 90 sec after (right) ISO (1 μmol/L) addition at 30 sec (20× magnification, CFP excitation, YFP emission); increased cAMP generation in response to ISO decreases YFP emission, right panel. βAR-selective antagonist impact on cAMP generation was assessed via ICUE3 assay, where only very small effects of Prop (B), ICI (C), and CGP (D) were detected. Tracings are mean ± SEM (N = 3). [file prp20002-e00024-SD1.pdf]

A

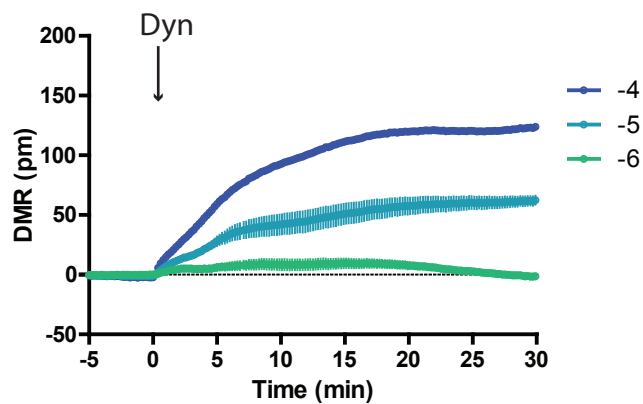

D

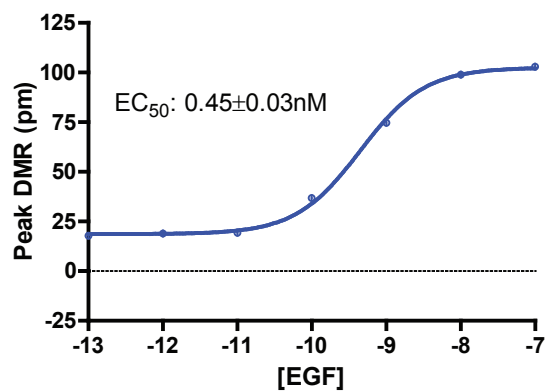

B

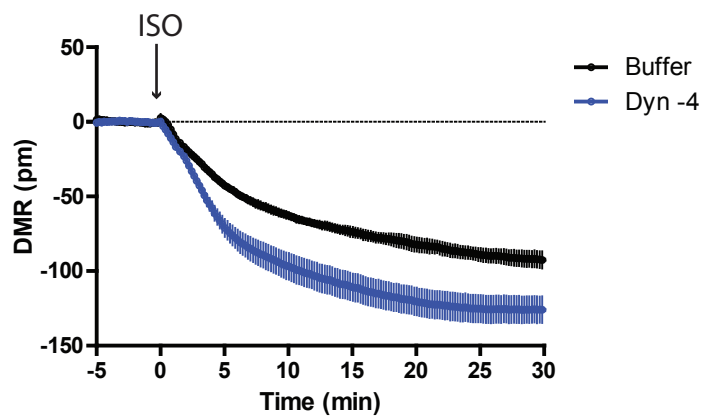

E

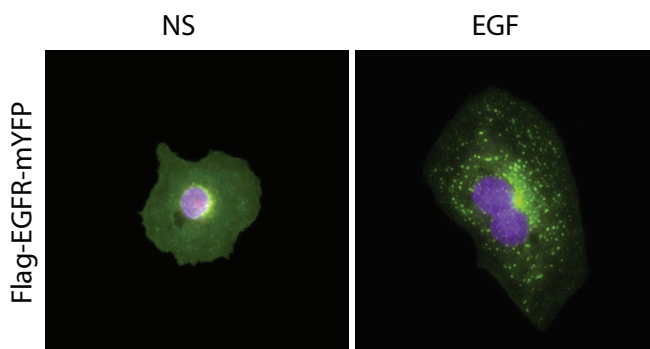

C

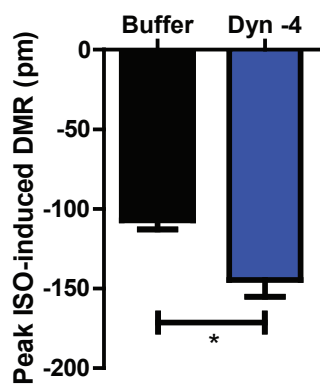

F

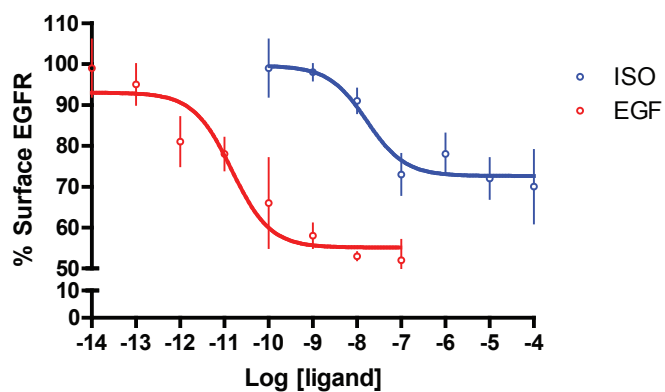

Supplemental Figure 2

Supplement: Supplementary file 2 — Figure S2. Dynasore and EGF signaling responses in NRCF. (A) The dynamin inhibitor dynasore (Dyn) had little impact on DMR responsiveness in NRCF at 1 μmol/L, but at or above its reported IC50 of 15 μmol/L, it induced positive DMR deflections. (B) Dyn pretreatment (100 μmol/L, 30 min) enhanced the ISO-mediated DMR effect. Tracings are mean ± SEM (n = 3). (C) Summary of peak ISO-induced DMR response from (B). Data are mean ± SEM (n = 3). *P < 0.05, two-tailed t-test. (D) EGF concentration dependently induced a positive-deflected DMR response in NRCF with an EC50 < 1 nmol/L. Data are mean ± SEM, n = 3 per concentration point. (E) EGF (1 nmol/L; 30 min treatment) induces the internalization of adenovirally expressed Flag-EGFR-mYFP in NRCF. Green = EGFR, blue = DRAQ5 nuclear stain. (F) Summary of on-cell assay for loss of surface Flag-EGFR-mYFP in response to increasing concentrations of EGF and ISO treatment for 60 min. Data are mean ± SEM, n = 3 per concentration point. [file prp20002-e00024-SD2.pdf]
